# FPGA-Based Smart Sensor for Online Displacement Measurements Using a Heterodyne Interferometer

**DOI:** 10.3390/s110807710

**Published:** 2011-08-05

**Authors:** Luis Alberto Vera-Salas, Sandra Veronica Moreno-Tapia, Arturo Garcia-Perez, Rene de Jesus Romero-Troncoso, Roque Alfredo Osornio-Rios, Ibrahim Serroukh, Eduardo Cabal-Yepez

**Affiliations:** 1 HSPdigital, CA Procesamiento Digital de Señales, CA Telematica, DICIS, Universidad de Guanajuato, Carr. Salamanca-Valle km 3.5+1.8, Palo Blanco, 36885 Salamanca, Gto., Mexico; E-Mails: lavera@hspdigital.org (L.A.V.-S.); svmoreno@hspdigital.org (S.V.M.-T.); troncoso@hspdigital.org (R.J.R.-T.); ecabal@hspdigital.org (E.C.-Y.); 2 HSPdigital, CA Mecatronica, Facultad de Ingenieria, Campus San Juan del Rio, Universidad Autonoma de Queretaro, Rio Moctezuma 249, 76807 San Juan del Rio, Qro., Mexico; E-Mails: raosornio@hspdigital.org (R.A.O.-R.); ibrahim@uaq.mx (I.S.)

**Keywords:** smart sensor, FPGA, heterodyne interferometer, phase measurement

## Abstract

The measurement of small displacements on the nanometric scale demands metrological systems of high accuracy and precision. In this context, interferometer-based displacement measurements have become the main tools used for traceable dimensional metrology. The different industrial applications in which small displacement measurements are employed requires the use of online measurements, high speed processes, open architecture control systems, as well as good adaptability to specific process conditions. The main contribution of this work is the development of a smart sensor for large displacement measurement based on phase measurement which achieves high accuracy and resolution, designed to be used with a commercial heterodyne interferometer. The system is based on a low-cost Field Programmable Gate Array (FPGA) allowing the integration of several functions in a single portable device. This system is optimal for high speed applications where online measurement is needed and the reconfigurability feature allows the addition of different modules for error compensation, as might be required by a specific application.

## 1. Introduction

In the recent evolution of different industrial processes, the measurement of small nanometric scale displacements demands metrological systems of high accuracy and precision, even in the toughest environments. Interferometry-based displacement measurements, particularly those using the dual-frequency laser feedback effect interferometer considered in [1], have become the main instrument employed for traceable dimensional metrology. Displacement measurement systems based on this technique usually achieve a resolution of 79.1 nm. However, higher resolution is required in the most recent high-precision measurement applications. In this context increasing the resolution of nanometric measurements remains an open problem [2]. On the other hand an acceptable measurement speed is also desirable in modern applications [3,4] and the use of high-speed electronic systems can solve this necessity.

To achieve the desirable measurement resolutions the performance of the interferometric system has to be improved by reducing the effects of any factors that contribute to the noise-level increase and by eliminating system deviations caused by environmental variables, which become more significant at high resolutions. These factors can be reduced by improving the interferometer optical configuration or by compensating the external effects into the electronic measurement system, as already noted in [5]. Other proposals differ from this methodology and use image processing methods [6,7], but with a considerable increase of the processing time.

More recent approaches to improve measurement systems include those based on modification of the physical characteristics of the interferometric system, as in the case of [8], where a system based on the principle of total internal reflection (TIR) and surface plasmon resonance (SPR) using a right-angle prism with a four-layer device [prism-titanium(Ti)-gold(Au)-air] was used to reach resolutions of 0.45 nm. However, the displacement range is limited to ±500 nm and the results are unsuitable for most industrial applications. Also in [9] a new enhanced interferometer setup based on a mechanically and thermally, highly-stable glass ceramic was designed. The noise level remains below 5 pm/√Hz and it is useful for offline applications since it operates at a frequency above 0.01 Hz. Other proposals focus on compensating and controlling the variables which affect the physical characteristics of the optical system. For example, in [10] a high-resolution and high-accuracy dilatometer using a heterodyne interferometer is developed, but since the thermal effects are controlled in a vacuum chamber to measure the coefficient of thermal expansion, the system cannot be considered portable. Other methodologies have been implemented in which different resolutions are obtained from interferometric systems. In this context, Simon *et al.* [5] presented a real-time high resolution interferometer. The system achieves a resolution of 20 nm at speeds of 2 m/s based in a homodyne laser interferometer. This type of interferometer shows some disadvantages compared to heterodyne systems, mainly the impossibility to measure continuously. Zhang *et al.* [2] presented a system for displacement measurements based on a microcontroller and a field programmable gate array (FPGA) with a theoretical resolution of 0.791 nm. Since the processing is done by separate devices, the processing speed is greater than 32 μs, which is considered unsuitable for online applications. Other systems improve the sensitivity, but greatly decrease the speed of measurement. This is the case of document [11], where an all-digital phasemeter for precision length measurements using heterodyne laser interferometry is presented. The developed sensitivity is on the order of 0.5 pm/√Hz, but at frequencies of 10 Hz, which make the system unusable for online applications. Although high resolution interferometric systems have been developed the speed of these systems is slow, making them effective only for applications where online measurements are not required. For online positioning control a nano-positioning system using pulse wide approach with a reported precision of ±20 nm was proposed in [12]. A digital signal processor (DSP) is used as a control unit, but error compensation for environmental variables was not considered. Other proposed systems require specific environmental conditions and therefore cannot be implemented in all the areas of interest. Furthermore commercially available interferometric systems are typically used to measure displacements in air [13].

One of the main disadvantages of the works previously discussed lies in the lack of online processing capability. Interferometric systems require complex processing to compensate and reduce the effects of uncertainty [14], then it is essential to carry out an on-line processing of the variables involved and to include self-adjustment algorithms. This requires the use of intelligent and smart sensors which are capable of processing measured variables in an integrated system where this feature can be performed according to the definition of smart sensor [15]. The use of smart sensors in industrial applications that require online monitoring has been extensively studied, and due to the high processing capability required they have been implemented into an FPGA, for example for motion dynamics estimation based on an encoder sensor [16], tool-wear monitoring using accelerometers [17], and the fusion of these sensors for complex estimation of tool-wear [18], also for mechatronics there are sensors based on interferometry reported in [19], such as an optomechatronic sensor for the detection of multi-degrees-of-freedom displacements of a remote target.

Nowadays nanometric motion quality, which is defined in terms of precision, accuracy, and resolution, are vital to several existing and emerging nano-scale microscopy, manipulation, and manufacturing methods, with less than 10 nm of accuracy [20], since it comprises sensors and electronics, nanometric metrology systems are essential. For instance, in metallographic analyses there are several necessities in terms of motion quality [21], summarized in Table 1.

The contribution of this work is the development of a smart sensor based on phase measurement achieving high accuracy and high resolution on the order of nanometers that is designed to be used with a heterodyne interferometer. The phase measurement is made by combining two techniques to measure the fractional phase into an interference light fringe. The first technique is based on digital counting and the second on analog-to-digital conversion and processing. Since both techniques require complex processing capabilities, the measurement system is implemented in a low-cost FPGA. The novelty of this work is the fusion of these techniques and the implementation in an FPGA allowing the system to achieve high speed measurements, on the order of MHz, therefore the system is optimal for high speed and for a wide range of measurement applications, while the reconfigurability feature allows the addition of different modules for error compensation as required by each application. In this case a self-wavelength deviation compensation is implemented to achieve better accuracy; furthermore the phase measurement system is low-cost and portable.

## 2. Interferometry

Optical interferometry is considered an optical path differences measurement technique and its bases were mainly established by Michelson in the last decades of the nineteenth century. Displacement interferometry is usually based on the Michelson configuration or some variant of that basic design. Displacement measurement, being simply a change in length, is usually carried out by counting the number of fringes as the object being measured is displaced. The displacement is measured as an integer number of whole fringes and a fringe fraction [22].

**Figure 1.**
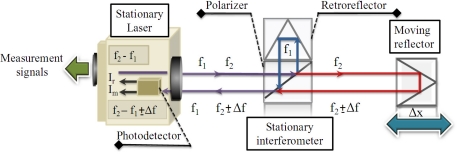
Heterodyne laser system operation.

Homodyne and heterodyne are two different methods of interferometric systems depending on the type of the laser used. A single frequency laser source is used in homodyne systems and for heterodyne systems, there are two laser beams with two different frequencies generating different methods: Acousto-optical modulation (AOM) and Zeeman effect. Figure 1 shows a heterodyne laser system where the beat laser beam before entering the interferometer is proportional to the next signal as a function of the difference in frequency and phase of the two beams that are perpendicular as shown in Equation (1) where *E_1_* and *E_2_* represent the amplitude of the beams, *f_1_* and *f_2_* represent their frequencies and *φ_1_* and *φ_2_* are they respectively phases [23]:

(1) $$I_{r}=2E_{1}E_{2}\text{cos}[2\pi (f_{2}-f_{1})t+(\phi_{1}-\phi_{2})]$$

This first beat signal is named reference signal *I_r_*, being very stable in frequency and equal to the difference in frequency between the beams. If at the laser output the beam is divided, each beam travels by different paths into the interferometer, one to the fixed reflector and the other to the moving reflector. Both laser beams are physically combined and after passing through a polarizer, in a second photodetector its beat is detected as shown in Equation (2) where *φ_m_* and *φ_r_* are the phases of measurement and reference signals, respectively:

(2) $$I_{m}=2E_{1}E_{2}\text{cos}[2\pi (f_{2}-f_{1})t+(\phi_{1}-\phi_{2})+(\phi_{m}-\phi_{r})]$$

*I_r_* and *I_m_* differ only in one phase, which is proportional to the difference on paths recorded by each beam. This second signal is named measurement signal, being its phase and frequency variable during the displacement of the moving reflector. When the moving reflector moves, this difference in phase depends on the time, creating a Doppler shift of the frequency of the second beam that is proportional to the velocity *v*. The difference in frequency Δ*f* is given by Equation (3) where *f_2_* is the measurement beam frequency, *c* is the speed of light and *n* represents the number of cycles shifted:

(3) $$\Delta f=\frac{2{\mathrm{vnf}}_{2}}{c}$$

The difference in phase between reference and measurement signals is the displacement of the moving reflector according to Equation (4) where *Δx* is the resulting displacement, *λ_2_* is the wavelength of the measurement laser beam and *Δφ* is the difference in phase [22,23]:

(4) $$\Delta x=\frac{\lambda_{2}}{4\pi n}\Delta \phi$$

## 3. Methodology

This section shows the methodology followed for the smart sensor development and the characteristics and capabilities of the system. First the smart sensor general structure will be discussed, and then the architecture of the FPGA-based processor is presented.

### 3.1. FPGA-Based Displacement Measurement Smart Sensor

**Figure 2.**
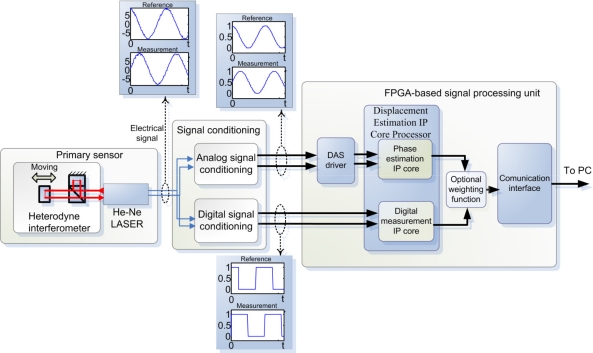
Schematic diagram of the smart sensor proposed.

The schematic diagram of the displacement measurement smart sensor is shown in Figure 2. The implemented system consists of three units: primary sensor, signal conditioning and FPGA-based signal processing unit. The primary sensor includes a commercial He-Ne laser head and an optical heterodyne interferometer and the laser head includes an integrated photodetector which produces an electric output for the measured beam and for the reference beam as well. The signal conditioning includes two stages: digital and analog signal conditioning. The digital stage provides the appropriate conditioning of the laser signals, a digital driver changes the voltage level of the analog signal, a high speed Schmitt trigger inverter changes the signal to the appropriate voltage level with the same path delay for both the reference and measurement signals. The analog signal conditioning includes a high speed dual channel data acquisition system (DAS) with low distortion differential amplifiers to allow the minimum noise distortion of the signals received from the laser. The FPGA-based signal processing unit contains a measurement IP core to estimate the displacement of the mirror by counting the number of cycles forward or backward from the measurement signal with respect to the reference signal. This unit also contains a phase estimation IP core to determine the phase between both signals together with a DAS driver. This processing unit also can include interfaces for displaying and user communication.

### 3.2. Displacement Estimation IP Core Processor

**Figure 3.**
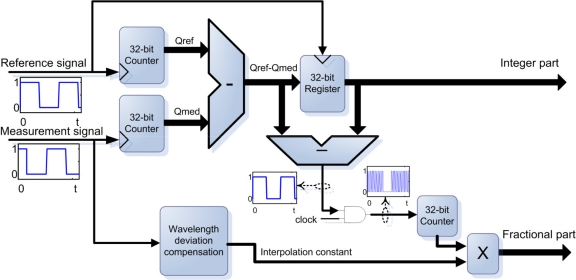
Block diagram of the digital measurement IP core.

The central part in the processing unit is the measurement and displacement estimation performed in an IP core. The system is divided in two IP cores working separately, the first IP core is an all-digital count fringe system, shown in Figure 3. This core counts the total cycles of both signals, reference and measurement. When the measurement changes its frequency the counter is delayed or advanced and the difference of counters provides the amount of fringes displaced. During a cycle this difference changes due to the signals phase, so in order to maintain the current count a 32-bit register stores the data at the beginning of the cycle. This architecture gives an exact count of the total fringes in real time. To estimate the fractional part, a difference between the actual count and last difference counter is performed, this difference results in a 1-bit modulated signal which represents the phase in PWM (Pulse Width Modulation) with the same beat frequency. To determine the duty cycle of the modulated signal that is proportional to the phase, a demodulator system is implemented. The demodulator is based on a dual clock edge counter, the counter is active during the high level of PWM signal, an AND gate is used for this purpose, then the count is synchronized with the measurement signal due to a parallel register. A processing block performs the correct estimation of the displacement depending of the present frequency of the input signals. This displacement compensator measures the signal frequency of each cycle to compensate the wavelength variations as established in [13], although several blocks can be implemented in order to compensate other errors due to reconfigurable capabilities of the system.

**Figure 4.**
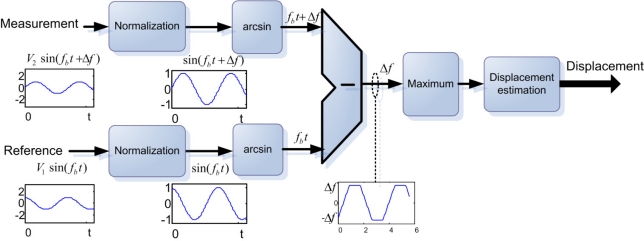
Block diagram of the phase estimation IP core.

The second IP core is based on a phase estimation (A block diagram of this system is presented in Figure 4). The analog signal is digitalized by a 14-bit dual-flash ADC at 20 MHz, *Δf* represents the frequency difference and, *V_1_* and *V_2_* the amplitude of the signals. The converted data is normalized in order to extract the inverse sine function; this function is calculated by a simple look-up table (LUT) and the result is the approximation of the angle of each signal. The measurement and reference signals have different phase but the same frequency, so the angle difference represents the phase along each cycle. In a complete beat frequency cycle there are up to seven conversions from the ADC, for these processed samples it is necessary to determinate the maximum angle to discriminate the zero computed phase when both signals cross in time. When the phase is calculated, a displacement estimation process is performed where some compensation can be reconfigured if the application demands more stability. For this estimation the resolution depends on the number of samples in a beat frequency cycle and on the precision of the inverse sine function.

The resulting displacement is obtained by both methodologies calculating a value from a simple weighted function of the displacement. The trigonometric estimation calculates the displacement with certain delay that depends on the ADC conversion time and trigonometric calculation, the total delay of this part is 414 ns. To allow the correct measurement it is necessary to add a control module for delaying the digital estimation in order to synchronize the measured data in time. For steady measurements the displacement can be processed with a digital filter in order to reduce the noise level.

The theoretical resolution of this system depends on the laser wavelength *λ*, the beat frequency *f_b_* and the digital system clock frequency *f_clk_*. The beat frequency is estimated by the difference of the two polarized beam frequencies as follows:

(5) $$f_{b}=f_{2}-f_{1}$$

To determine the resolution the maximum number of clock edges that can be count over a period of the beat frequency must be calculated. This is done by dividing the double clock frequency by the beat frequency, this is:

(6) $$Nc=\frac{2f_{\mathrm{clk}}}{f_{b}}$$

Then the theoretical resolution *ρ* is the ratio of the distance per beat period *λ/2* and the maximum number of clock counts *Nc*:

(7) $$\rho =\frac{\lambda }{2\mathrm{Nc}}$$

For this system the theoretical resolution is 3.40 nm according to Equation (7), for a laser wavelength of 632.9911354 nm, a beat frequency of 2.688 MHz and a clock frequency of 125 MHz.

## 4. Experimental Section

In this section, the experimental setup and the results for validating the proposed smart sensor are presented.

### 4.1. Experimental Setup

**Figure 5.**
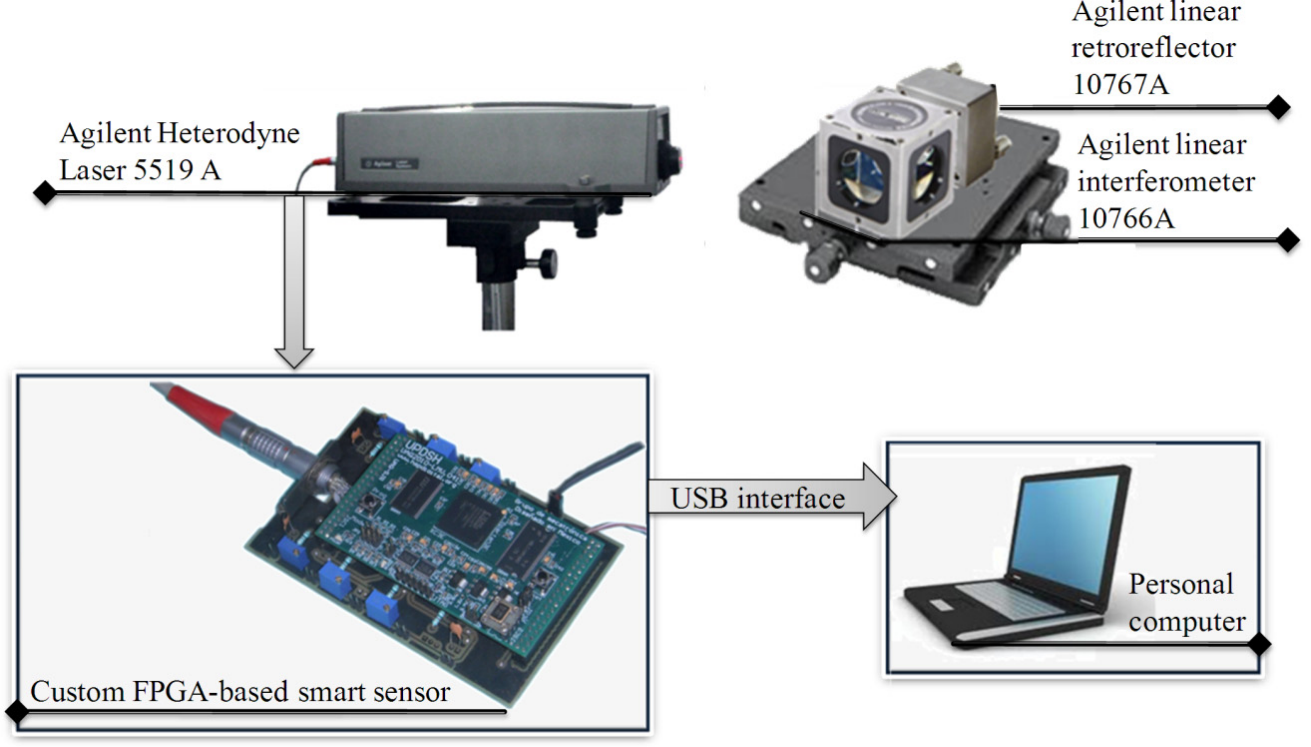
Block diagram of experimental setup.

The experimental setup is shown in Figure 5, which displays the arrangement of the optics for both experiments. A calibrating system is used as primary sensor, which contains a two-frequency laser calibrator 5519A with vacuum wavelength of 632.991354 nm, a linear interferometer 10766A with a fundamental optical resolution of λ/2 (316.5 nm) and two linear retroreflectors 10767A, all from Agilent Technologies [24]. The data acquisition is performed by a 14-bit 2-channel AD9248 ADC from Analog devices with maximum sampling rate of 20 MHz [25]. The signal compensation module uses four low distortion differential ADC drivers AD8138 both from Analog devices [26]. The signals obtained from the laser are sent to the smart processor to estimate the linear displacement. The smart processor is implemented in a proprietary Spartan 3E XC3S1600E FPGA platform running at 125 MHz developed by the authors. A communication interface unit is added to the smart processor in order to send the monitored signals to a personal computer to be visualized and processed for analysis.

### 4.2. Experimentation, Results and Discussion

Two experiments to validate the maximum resolution and the accuracy were performed and are presented in this section. In the first experiment the resolution of the smart sensor is evaluated using a calibrated pattern and a metallographic microscope. In the second experiment the accuracy of the system is determinate by measure the displacement of the material due to the linear thermal expansion. The results of the proposed experiments are presented and discussed; also the main advantages of the proposed methodology are analyzed.

### 4.2.1. Precision

**Figure 6.**
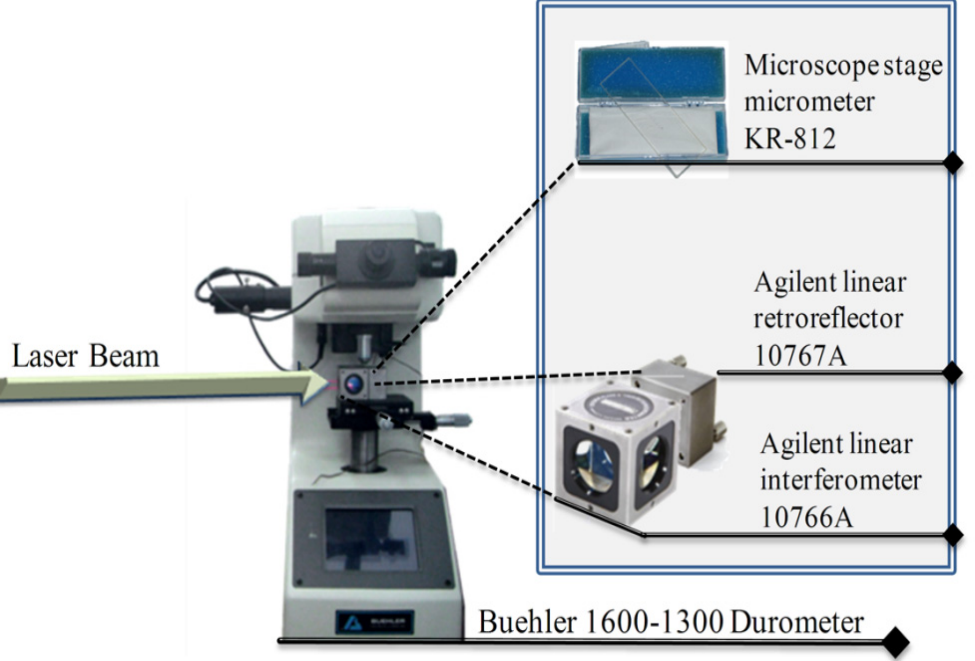
Moving reflector and calibration pattern mounting.

In this experiment the precision of the smart sensor is evaluated by using a stage micrometer KR-812 with NIST (National Institute of Standards and Technology) traceable calibration from microscope depot with a minimum scale of 20 μm. Figure 6 shows the moving reflector and the calibration pattern mounted on a Micromet 1,600–1,300 durometer from Buehler [27].

**Figure 7.**
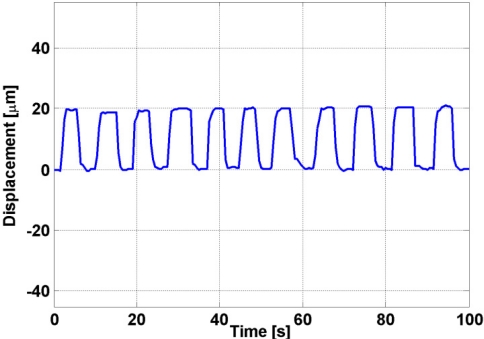
Signal measured for 20 μm displacements.

Using the stage micrometer as reference in this experiment movements of 20 μm were performed as shown in Figure 7.

Forty measurement runs were repeated in order to have enough tests for determining the system precision, obtaining a mean μ = 20.03 μm and a standard deviation σ = 0.02317 μm. For a 99.73% certainty, in a 20 μm displacement, the system has a precision of 3σ = 0.06950 μm.

### 4.2.2. Accuracy

To measure the system accuracy the displacement in the order of nm has to be performed using the thermal expansion of the mirror base. The linear strain is defined relative to some reference distance *L̄*. Ideally the linear strain is defined in Equation (8), where *ε* is the linear strain and *δL* is the difference of displacement [28]:

(8) $$\varepsilon =\int_{L}^{L}\frac{\mathrm{dL}}{L}=\ell n\left(\frac{L}{\bar{L}}\right)=\ell n\left[1+\left(\frac{\delta L}{\bar{L}}\right)\right]$$

For a temperature derivative the coefficient of linear expansion *α* can be calculated by using Equation (9) where *δT* can be expressed as the difference of temperature *T_2_–T_1_*:

(9) $$\alpha =\left(\delta \ell n\frac{L}{\delta T}\right)=\left(\frac{1}{L}\right)\left(\frac{\delta L}{\delta T}\right)=\left(\frac{\delta L}{L}\right)\left(\frac{1}{T_{2}-T_{1}}\right)$$

**Figure 8.**
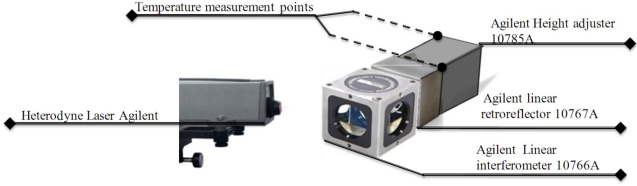
Optical arrangement for linear expansion measurement.

Experimentally, the expansion coefficients are usually obtained by measuring the dilation over a finite temperature interval, Figure 8 displays the optical arrangement for this experiment. Height adjuster (10785A) and the linear retroreflector (10767A) are made of stainless steel (416) from Agilent Technologies. The linear thermal expansion coefficient is 9.9 ppm/°K given by the manufacturer.

**Figure 9.**
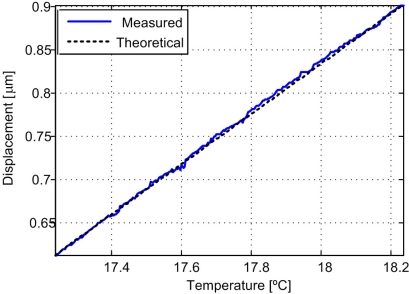
Graphic of displacement *vs.* temperature in accuracy experiment.

For temperature measurements a Fluke 52II dual input thermometer with an accuracy of 0.05% was used [29]. The material was cooled to a temperature of 13 °C until stabilization was achieved for measuring the temperature at two extremes of the material. Then measurements were made for displacement and temperature during warming by room temperature. Figure 9 displays a graphic of the displacement by the linear expansion during warming from 16.2 °C to 18.3 °C.

The theoretical linear expansion is calculated using Equation (9) resulting in an expansion of 0.0247 μm per 0.1 °C. For this experiment the resulting displacement derivative is μ = 0.0279 μm and σ = 0.0115 μm for 256 measurements. The accuracy of the system for a maximum mean error is 9.9 nm for a measured range of 293 nm.

### 4.3. Methodology Comparative

In order to validate the main advantages of the proposed system to other similar developed systems, a comparison of the main features on each system as resolution, accuracy among others, is shown in Table 2.

The methodology to determine the accuracy in [2] is not reported, nevertheless in this experiment the average accuracy using linear thermal expansion is limited by the temperature measurement and some environmental factors that can be reduced with compensation modules.

## 5. Conclusions

The smart sensor based on phase measurement developed in this work has the capability to measure in the nanometric range using a heterodyne interferometer. The novelty of this work is the fusion of the digital count and the analog to digital conversion techniques which provides a resolution of 3.4 nm, an accuracy of 9.9 nm and a precision of 69.5 nm in a 20 μm displacement for long range displacements. Furthermore, the comparison of reported methodologies shows that this proposal has the lowest processing time within 357 ns and the widest range of measurement. Although most works do not report the accuracy, the developed system is better than the references that report accuracy. The range of measurement that this system can perform is set by the optics, in this case the limit is 80 m; however, the system is capable to process measurements at greater distances with the appropriate optics and with a resolution of 3.4 nm. Tests performed in the experiment where made within a 3 m displacement range which is suitable for most of industrial applications. Moreover, additional modules for error compensation can be added to the proposed FPGA-based smart sensor as the application demands. Since the system is portable and because all the features gathered together, it is optimal for most industrial applications.

## Figures and Tables

**Table 1.** Metallographic typical applications requirements.

**Feature**	**Typical application**	**Requirement**	**Description**

Resolution	Surface topography	1–400 μm	Roughness differences can be measured, which is useful in examining machined surfaces and for measurement of surfaces layers or films.
Distance	Large Area Disector (LAD) technique	>300 mm	Metallographic better-quality data obtained from analyzing a larger sample area and/or more samples.
Accuracy	Inclusions in a heat of steel	30 μm	Distance between two fields of view is about 30 μm
Precision	Hardness test	20 μm–1 mm	Metallographic automatic stage movement ensures selection of fields without introducing operator bias.
Velocity	Number Density of microstructural features	0.2 m/s	For obtaining about 25 to 100 microstructural fields, high speed on sampling is required.
On-line	Metallographic automatic stage	<1 ms	Automatic devices permit more rapid data collection; image analyzers can perform several measurements on field within milliseconds.

**Table 2.** Features comparative between the proposal and reported works.

**Reference**	**Interferometer type**	**Resolution**	**Accuracy**	**Portability**	**Online (processing time)**	**Range of measurement**

Zhang *et al.* [2]	Heterodyne	0.791 nm	40 nm	YES	YES (32 μs)	<1 mm
Simon *et al.* [5]	Homodyne	20 nm	*	NO	YES (*)	*
Schuldt *et al.* [9]	Heterodyne	5 pm/√Hz	*	NO	NO (100s)	*
Wang [8]	Heterodyne	0.45 nm	*	NO	YES (*)	±500 nm
Zelenika and De Bona [12]	Michelson	10 μm	*	NO	YES (Offline identification)	1 mm
This work	Heterodyne	3.4 nm	9.9 nm	YES	YES (357 ns)	>3 m

* Not reported
